# Systemic right ventricular fibrosis detected by CMR predicts adverse clinical outcome in patients after atrial redirection surgery for transposition of the great arteries

**DOI:** 10.1186/1532-429X-17-S1-O94

**Published:** 2015-02-03

**Authors:** Riikka Rydman, Michael A Gatzoulis, Philip J Kilner, Dudley J Pennell, Sonya V Babu-Narayan

**Affiliations:** 1 Clinical Physiology, Molecular medicine and surgery, Karolinska Institute, Stockholm, Sweden; 2 NIHR Cardiovascular Biomedical Research Unit, Royal Brompton Hospital, London, UK; 3 Imperial College London, National Heart and Lung Institute, London, UK

## Background

The cross-sectional nature of our previous study by late gadolinium enhancement (LGE) cardiovascular magnetic resonance (CMR) in systemic right ventricle (RV) patients limited our ability to make conclusions about the prognostic predictive value of LGE in this population. Therefore, we performed this larger prospective study to determine whether fibrosis detected by LGE CMR would predict adverse outcomes in patients treated for transposition of the great arteries by atrial redirection surgery with RV in the systemic position and at risk of arrhythmia, premature RV failure, and sudden death.

## Methods

Fifty-five patients (aged 27±7 years) underwent LGE CMR and were followed for a mean of 6.5±3.2 years in a single-centre study. The pre-specified composite clinical endpoint consisted of new-onset sustained atrial/ventricular tachyarrhythmia or decompensated heart failure admission/transplantation/death.

## Results

RV LGE was present in 31 (56%) patients. In 8 out of 9 (89%) patients with more than one event, atrial tachyarrhythmia, itself a known risk factor for mortality, occurred first. Univariate predictors of the composite endpoint (n=22 patients; 19 atrial/2 ventricular tachyarrhythmia, 1 death) included RV LGE presence and extent, RV volumes/mass/ejection fraction, right atrial area, peak VO_2_ and age at repair. In bivariate analysis, RV LGE presence was independently associated with the composite endpoint, and remained significant (hazard ratio (HR) 4.95[95% confidence interval (CI) 1.60-15.28],p=0.005) when compared with the second independent predictor, percent predicted peak VO_2_ (HR 0.80[95% CI 0.68-0.95],p=0.009 per 5%). When fibrosis status was added to peak VO_2_%, risk prediction for cardiac events was further refined.

There was clear agreement between location and extent of RV LGE at *in vivo* CMR and histologically documented focal RV fibrosis in an explanted heart from one of the study patients. Lastly, RV LGE appeared progressively extended in another patient from our cohort restudied for clinical indication.

## Conclusions

LGE in the systemic RV was commonly found in our cohort of adult patients late after atrial redirection surgery for transposition of the great arteries and is a strong and independent predictor of adverse clinical outcome. RV LGE should therefore be incorporated in risk stratification for adverse cardiac outcomes in these patients.

## Funding

Riikka Rydman was supported by the Swedish Society of Medicine, Swedish Heart-Lung Foundation and Swedish Society for Medical Research and by the Section of Clinical Physiology, Department of Molecular Medicine and Surgery, at Karolinska Institutet, Stockholm, Sweden. Sonya V. Babu-Narayan is supported by an Intermediate Clinical Research Fellowship from the British Heart Foundation (FS/11/38/28864). This project was supported by the National Institute for Health Research (NIHR) Cardiovascular Biomedical Research Unit of Royal Brompton and Harefield National Health Service (NHS) Foundation Trust and Imperial College London. This report is independent research by the NIHR Biomedical Research Unit Funding Scheme. The views expressed in this publication are those of the author(s) and not necessarily those of the NHS, the National Institute for Health Research or the Department of Health.

